# Movement patterns of cheetahs (*Acinonyx jubatus*) in farmlands in Botswana

**DOI:** 10.1242/bio.021055

**Published:** 2016-12-02

**Authors:** L. K. Van der Weyde, T. Y. Hubel, J. Horgan, J. Shotton, R. McKenna, A. M. Wilson

**Affiliations:** 1 Cheetah Conservation Botswana, B5, Kgale Siding Office Park, Plot 1069-KO, Gaborone, Botswana; 2 San Diego Zoo, Institute for Conservation Research, 15600 San Pasqual Valley Road, Escondido, CA 92027-7000, USA; 3 Structure and Motion Laboratory, Royal Veterinary College, University of London, Hatfield AL97TA, UK

**Keywords:** T-LoCoH, Home range, Resource selection, Non-protected, Human-wildlife conflict

## Abstract

Botswana has the second highest population of cheetah (*Acinonyx jubatus*) with most living outside protected areas. As a result, many cheetahs are found in farming areas which occasionally results in human-wildlife conflict. This study aimed to look at movement patterns of cheetahs in farming environments to determine whether cheetahs have adapted their movements in these human-dominated landscapes. We fitted high-time resolution GPS collars to cheetahs in the Ghanzi farmlands of Botswana. GPS locations were used to calculate home range sizes as well as number and duration of visits to landscape features using a time-based local convex hull method. Cheetahs had medium-sized home ranges compared to previously studied cheetah in similar farming environments. Results showed that cheetahs actively visited scent marking trees and avoided visiting homesteads. A slight preference for visiting game farms over cattle farms was found, but there was no difference in duration of visits between farm types. We conclude that cheetahs selected for areas that are important for their dietary and social needs and prefer to avoid human-occupied areas. Improved knowledge of how cheetahs use farmlands can allow farmers to make informed decisions when developing management practices and can be an important tool for reducing human-wildlife conflict.

## Abstract (precis)

**Summary:** Cheetah movement patterns within farmlands revealed a preference for areas important for social interactions and avoidance of high risk areas. This knowledge can be useful for developing conflict mitigation strategies.

## INTRODUCTION

Cheetah populations continue to decline worldwide, despite conservation efforts and increasing knowledge of cheetah ecology. Key drivers of decline include habitat loss, disease and persecution, which is widespread in much of southern Africa (Purchase et al., 2007). In farming areas, where cheetahs have been shown to occur in relatively high densities (Klein, 2007; Marker et al., 2003), they must co-exist with humans to survive, but how they may adapt their movements and behaviours in these landscapes is not well understood. Knowledge of both broad-scale and fine-scale movement patterns that show selection or avoidance of both natural and man-made features is important for cheetah conservation in farming areas (Kent, 2011). Understanding potential adaptions to these environments may reveal how cheetah behaviour on farmlands may differ from that observed in the more commonly studied protected areas (Durant et al., 2004; Kelly and Durant, 2007). Insight on cheetah spatial ecology in farmlands will enable improved methods in human-wildlife conflict mitigation, a vital component of cheetah survival in these areas.

Botswana has the second largest population of cheetah and retains large connecting habitats across the country (Klein, 2007; Purchase et al., 2007) with 38% of the land set aside for wildlife utilisation (Winterbach et al., 2015). Cheetahs, like other large carnivores, have large home ranges, but despite several protected areas being large enough to accommodate these ranges, cheetahs exist at low densities in these areas (Klein, 2007) and are more commonly found in non-protected areas. Studies of cheetah home ranges have shown large variability in size, primarily as a function of prey availability, habitat, sex and interspecific competition (Caro, 1994; Durant, 2000). Early cheetah studies in large open protected habitats, such as the Serengeti National Park, revealed average home range sizes around 700 km^2^ for non-resident males and around 800 km^2^ for females (Caro, 1994). Conversely, in similar protected habitats in two fenced South African national parks, home ranges of less than 200 km^2^ for both sexes were recorded (Broomhall et al., 2003; Welch et al., 2015). In these cases, cheetah residency status or prey availability and their migratory patterns may be responsible for the differing home range sizes. The non-protected areas favoured by cheetahs in some regions may be due to the low or lack of presence of larger competitors such as lion (*Panthera leo*) and spotted hyaena (*Crocuta crocuta*). The presence of these competitors has been shown to affect temporal and spatial movement of cheetahs (Bissett et al., 2015; Cristescu et al., 2013; Durant, 2000), with cheetahs actively avoiding areas and moving away from larger competitors. Whilst cheetahs occupying areas with low interspecific competition might be expected to have smaller home ranges (as the need to avoid more competitive species is reduced), this is not always the case (Purchase and du Toit, 2000). Similarly, in Namibian farmlands with low densities of large carnivores (Marker et al., 2008a), the home range sizes of cheetahs are exceptionally large, averaging 1600 km^2^. Furthermore, the authors found that these large home ranges were not related to sex, social status or season. In farmlands of Botswana, male cheetah home ranges averaged 668 km^2^ (Houser et al., 2009a), smaller than those found in Namibia, but still considerably larger than the male home ranges of 190-310 km^2^ observed in South African ranches (Marnewick and Cilliers, 2006). These findings suggest that whilst the low density of large competitive carnivores in farmlands may have some effect on ranging patterns of cheetahs, prey availability may be more influential (Houser et al., 2009a).

Farming environments, such as those in the Ghanzi district of Botswana, generally contain resources such as permanent water sources necessary for livestock, and naturally occurring prey species which are attractive to predators (Marker-Kraus and Kraus, 1994; Winterbach et al., 2015). However, farmlands are also highly modified landscapes and movement of livestock or game is regularly managed, leading to regular human presence across the farms. Furthermore, infrastructure like the presence of homesteads (farm houses) and artificial barriers such as roads and fences will also likely affect wildlife movement, even if they do not directly impede it (Boast and Houser, 2012; Forman and Alexander, 1998; Hayward and Kerley, 2012). Prey availability may also vary according to farm type. Game farms have higher numbers of antelope species than cattle farms (Boast and Houser, 2012), and the medium-sized prey species preferred by cheetahs (Boast et al., 2016; Hayward et al., 2006) may also vary in density depending on farm type. The changing landscapes in farming areas due to presence of livestock and resultant overgrazing, increases in food and water availability, and reduced pressure from competitors (Mcvittie, 1979) are very likely to result in changes in cheetah behaviour and movement compared with those found in protected areas.

The objective of this study was to examine how cheetahs may have adapted their movement in these farmlands by using high-time resolution GPS collars that can record fine scale movements (Hubel et al., 2016a; Wilson et al., 2013). In particular, we set out to determine home range sizes of cheetahs in these non-protected areas and evaluate factors that may influence size. We also aimed to assess whether cheetahs selected or avoided particular natural resources, artificial structures, locations within farms and farm type by evaluating visitation and duration rates. Scent-marking trees (hereafter marking trees) were selected as a natural resource as they are highly sought out by cheetahs (Marker-Kraus and Kraus, 1994); homestead locations were selected as artificial structures; the centre points of farms were used as a location variable and we also compared cattle and game farms. To determine selection or avoidance we used a time-based local convex hull method that can be used to determine visitation and duration rates from GPS telemetry data (Lyons et al., 2013). The following hypotheses were tested: (a) cheetah home ranges are affected by residency status; (b) preferred natural resources will be visited more often and for longer periods than areas of human habitation; (c) location preferences will have an effect more on duration than visitation rates, and (d) visitation frequency and duration is higher on game farms than cattle farms.

## RESULTS

### Home-range analysis

Individuals cheetah used on average 21±6 s.d. farms (range 13–39), with individual M3 ranging over the greatest number (39) of farms. Average farm size was 68.7 km^2^±31 s.d. (range 12.4–272.5) with a total of 64 farms being visited by at least one collared cheetah. Of these, 20 farms were visited by at least two of the collared cheetahs. The average proportion of game farms (0.25±0.05 s.e.m.) used by the cheetahs was lower than the proportion of cattle farms (0.75±0.05 s.e.m.). Home range sizes varied between the four individual cheetahs (Table 1) depending on the method of home range analysis. Home range differences between individuals were expected as the period of collaring time varied between individuals. Based on existing knowledge of cheetahs seen on camera traps in the area, M1, M2 and M4 are considered resident males and M3 a non-resident. As our sample size was very small, it was not possible to conduct any statistical analyses comparing home ranges based on residency status; however, it is clear that the largest home range was that of the non-resident male despite having the shortest collaring period and the smallest number of fixes. The minimum convex polygon (MCP) method produced much higher home range estimates than either the kernel density estimator (KDE) or time-local convex hull (T-LoCoH) methods.

**Table 1.**
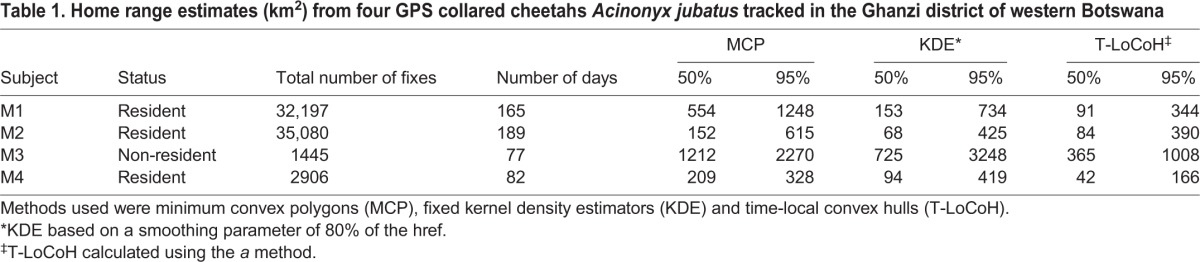
**Home range estimates (km^2^) from four GPS collared cheetahs *Acinonyx jubatus* tracked in the Ghanzi district of western Botswana**

### Multilevel meta-analysis

All models showed a significant amount of residual heterogeneity (high Q statistic, *P*<0.001) implying that the predictors we tested do not account for large amounts of between-individual variance in effect sizes. Despite cheetahs coming from the same population in this study, there were large differences in effect sizes between individuals. Estimated coefficients for average duration of visit and number of visits for each of the three distance predictors produced by each model are shown in Table 2.

**Table 2.**
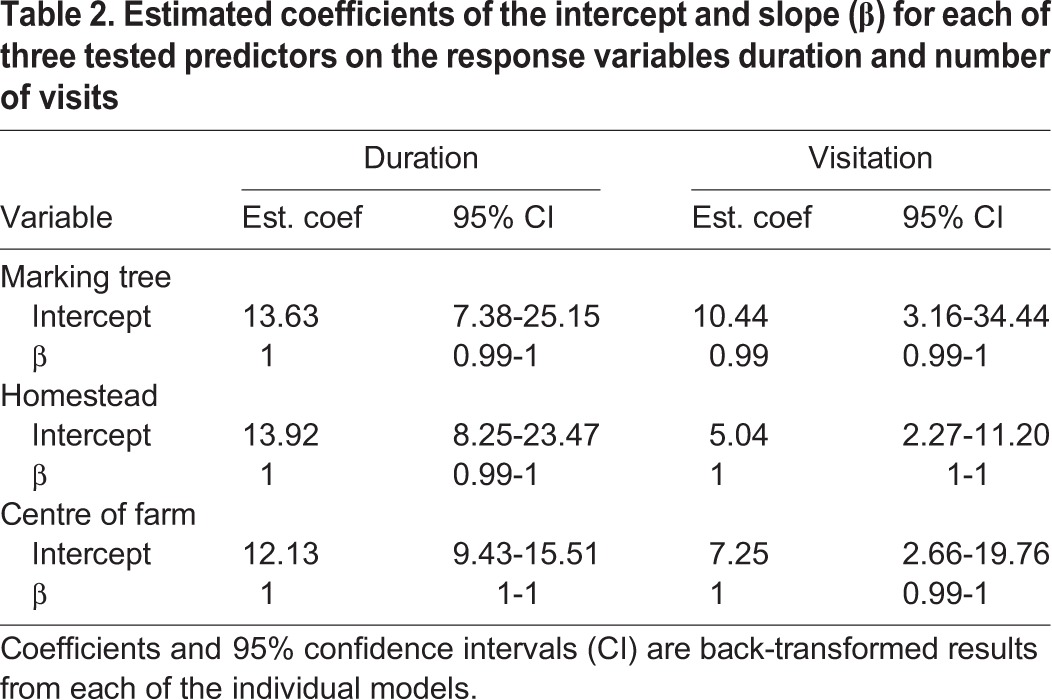
**Estimated coefficients of the intercept and slope (β) for each of three tested predictors on the response variables duration and number of visits**

### Visitation

**Fig. 1.**
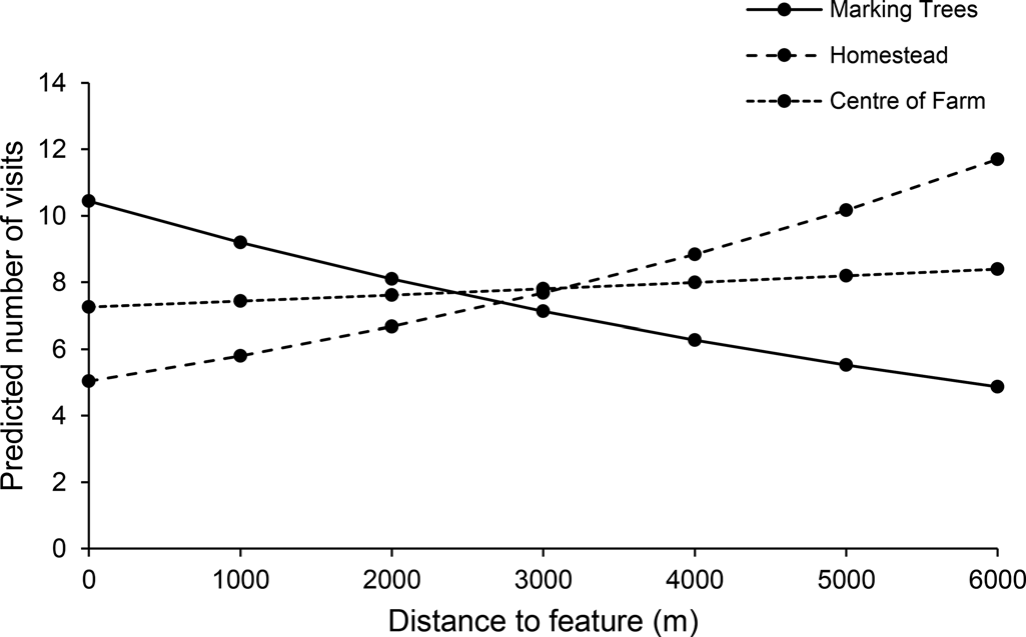
**The predicted number of visits of cheetahs *Acinonyx jubatus* (*n*=4) in response to the distance from marking trees, homesteads and central points of farms.** A two-stage multilevel meta-analysis with random effects was used to model the data. Visits to marking trees and homesteads were significantly affected by distance to these features (*P*<0.05).

When analysed separately, the number of visits were significantly affected by distance to marking trees (z=−2.40, *P*=0.02), with the highest number of visits in areas close to marking trees (Fig. 1). There was some degree of residual variability between subjects in number of visits at marking trees (τ^2^=1.48±1.22 s.d.). Distance to homesteads was significantly positively associated with visitation rates (z=6.44, *P*<0.01). Cheetahs visited areas further away from homesteads most often but showed variability between individuals in visits directly at homesteads (τ^2^=0.66±0.81 s.d.). Similarly, distance to centre of farms was also positively associated with visitation rates, but this was not significant (z=0.41, *P*=0.68). Residual variability between individuals on the number of visits at the centre of farms was high (τ^2^=1.04±1.02 s.d.). In each of these cases, all heterogeneity between subjects in the slopes was explained by the predictor as shown by τ^2^=0 in each case. In our global model analysis, where we incorporated all predictors associated with distance, only distance to marking trees had a significant negative effect (z=−3.31, *P*<0.01). There was also some degree of variability between individuals in visitation rates at marking trees (τ^2^=0.9).

Game farms were visited more often by cheetah than cattle farms, but this effect was not significant (z=1.58, *P*=0.11). The amount of unexplained variability between cheetahs was τ^2^=0.60 (±0.78 s.d.) for cattle farms and τ^2^=0.11 (±0.34 s.d.) for game farms, after accounting for farm type. The large standard deviations imply large differences between cheetahs in the number of visits.

### Duration

**Fig. 2.**
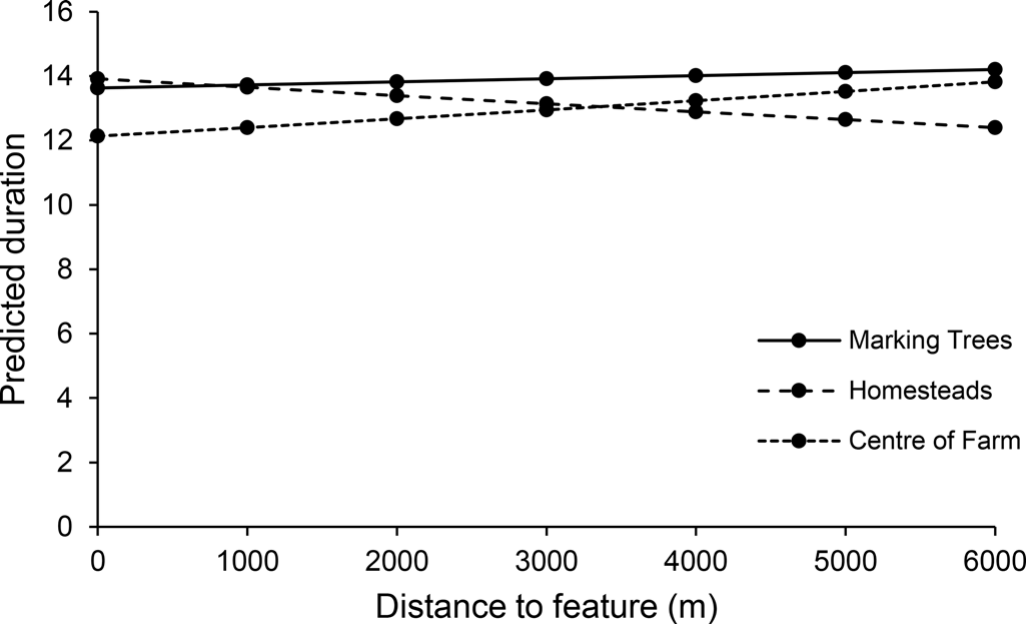
**Predicted duration of visit as a function of distance to marking trees, homesteads and central points of farms for four collared cheetahs (*Acinonyx jubatus*) in farmlands, Botswana.** Data were analysed using a two-stage multilevel meta-analysis. There was no significant effect of distance to each of these features on duration (*P*>0.05).

Periods of duration were not affected by any of the variables when tested independently in this study (Fig. 2). Distance to marking trees (z=0.20, *P*=0.84), homesteads (z=−0.46, *P*=0.65) and centre of farms (z=0.86, *P*=0.39) were all non-significant. In all cases the variability between individuals (τ^2^ below 0.4) and in the slopes (τ^2^=0) was low. However, when incorporaating all variables, distance to marking trees was significantly positively associated with duration rates (z=2.1, *P*=0.04), but neither distance to homestead or centre of farms had any effect. Variability was high between individuals (τ^2^=1.36).

Duration periods were not affected by farm type (z=−1.04, *P*=0.3), with cheetahs showing no preference for cattle or game farms. There was a larger amount of remaining residual variation between cheetahs on game farms (τ^2^=0.16±0.4 s.d.) than on cattle farms (τ^2^=0.02±0.13 s.d.).

## DISCUSSION

Cheetah home range sizes in this study may vary as a function of residency status, with a single non-resident cheetah showing a substantially larger ranging size compared with resident cheetahs. Our small sample size did not allow valid statistical conclusions for this effect, but it was not due to sex or differences in prey availability as all cheetahs' utilised similar proportions of game and cattle farms. Despite our relatively short collaring periods, due to the high resolution collars used, home ranges were similar to those in previous studies in farming areas in Botswana (Houser et al., 2009a), smaller than those reported in Namibia (Marker et al., 2008a), and larger than those in South Africa (Marnewick and Cilliers, 2006). Disparity in home range size across farming environments suggest that differing levels of available resources, such as water, prey and level of conflict, may be responsible for large scale variability (Marker et al., 2008a), but within specific regions other factors such as status may still be important.

Non-protected areas such as farmlands provide resources that are important for cheetah behaviour and habitat use. Marking trees were highly visited by all cheetahs when they were within an average daily distance of these. Marking trees are an important resource for male cheetahs. Males mark these trees through urination or clawing and this is considered to be a way to advertise a resident's territory (Eaton, 1970; Marker-Kraus and Kraus, 1994; Marnewick et al., 2006). Although cheetahs regularly re-visited marking trees, duration periods were similar or even shorter at these sites compared with areas further away from these sites. Similarly, duration period was not influenced by distance to homesteads, but as we expected, cheetahs avoided visiting homesteads. However, general spatial locations such as central points of farms did not influence either visitation or duration periods by cheetahs. Within the study area, cheetahs showed a slight preference for visiting game farms more often than cattle farms, despite the much lower proportion of game farms used by these cheetahs. This preference was not significant, most likely as a result of our small sample size and variability in visitation rates between cheetahs. Game farms generally have greater species-richness than cattle farms. Density and abundance of preferred prey species for cheetah in this area, such as duiker (*Sylvicapra grimmia*), steenbok (*Raphicerus campestris*) and greater kudu (*Tragelaphus strepsiceros*) (Boast and Houser, 2012; Boast et al., 2016) may vary depending on farm type. For example, the density of duiker has been found to be higher on cattle farms, whereas steenbok density was higher on game farms, but there was no difference for kudu (Kent, 2011). If prey availability is equally spread across both farm types, then this may explain similar duration periods found between cattle and game farms.

Other variables such as roads and fences are also likely to influence the movement of cheetah in farmlands. For example, roads have been shown to influence the movement of African wild dogs (*Lycaon pictus*). During travelling periods, particularly in dense environments, roads were actively used to enhance movement, but avoided during resting periods (Abrahms et al., 2015). However, roads have also been shown to negatively affect animal movements through avoidance by some species, increasing habitat fragmentation and mortality (Forman and Alexander, 1998). Although the extent of roads may be similar in farmlands with those in some protected areas, the additional construction of fences in farmlands is likely to provide another potential factor influencing movement. In one study, a single fence was shown to affect lion movement, but was more permeable to spotted hyaena, wild dog and cheetah (Cozzi et al., 2013). However, the high density of fences in farmlands and their effect on cheetah movement is unknown, as too few farms with adequate road and fence locations were available to be investigated in this study. Recent studies have shown important new insights into cheetah hunting strategies in protected areas (Wilson et al., 2013) and similar information is needed for cheetah in farmlands, where the landscape and habitat can vary considerably. Such information will provide valuable insight into understanding adapted hunting strategies in these environments, and highlight potential differences in hunting dynamics and kill rates between natural prey and livestock. This in turn may be useful in developing measures to mitigate livestock loss.

For cheetahs to co-exist in human-dominated environments, it is likely that they may need to adapt their movements to avoid human activity whilst still utilising areas that are important for their dietary and behavioural needs, as we have shown here. In order to help conserve cheetah and mitigate the human-wildlife conflict that is high in farmlands (Marker et al., 2003; Purchase et al., 2007), it is valuable to know what areas or resources may attract or deter cheetahs. The importance of marking trees to cheetahs has been exploited by researchers as sites that provide valuable monitoring and population assessment data (Marker et al., 2008b; Marnewick et al., 2008). However, marking trees have also been associated with negative attitudes and have been used by famers as a way to locate and remove cheetah (Marker et al., 2003; Mcvittie, 1979). In this study we have highlighted that cheetah actively visit these trees and this information can assist in helping farming management practices, such as adjusting movements of livestock or construction of kraals to avoid these areas. Similarly, as cheetah showed a preference for visiting game farms compared with cattle farms, maintaining high levels of small natural prey on these farms may help reduce conflict. A study assessing prey base for cheetah across Botswana found that maintaining a 20% wild prey biomass in farming areas can substantially reduce the potential for human-wildlife conflict (Winterbach et al., 2015). As non-protected areas such as farmlands provide valuable resources like prey, water and marking trees for cheetahs, knowledge of spatial movements can provide valuable information in understanding cheetah habitat utilisation, which can assist with improved measures that help people co-exist with these carnivores. Therefore, further studies on farmland cheetahs are needed to provide information to manage and protect high populations in these areas. This study has established some important characteristics of movement behaviour by cheetah, but there is still much to be learned to facilitate co-existence and protection of the species.

### Conclusions

As cheetahs continue to utilise and rely on non-protected areas, human-wildlife conflict will persist until we are better able to co-exist with predators. In this small-scale study, we were able to show detailed spatial movements in farmlands using high resolution GPS collars. This technology is relatively new and has not previously been used to detail fine scale movement patterns showing selection or avoidance of landscape features in these environments. Scent-marking trees have been shown to be a highly selected resource for males as shown by revisitation rates. Similarly homesteads are actively avoided as shown by low visitation rates to these areas. We found a slight preference for visitation to game farms compared with cattle farms, but duration rates were not affected by farm type. A better understanding of predator movements in highly human-dominated landscapes is necessary to assist with developing novel conflict mitigation measures and farm management practices, as a means to conserve these species.

## MATERIALS AND METHODS

### Area

The study was undertaken in the Ghanzi district, Botswana, an area consisting of communal and commercial farms as well as wildlife management areas (WMAs). The area is considered one of the most important areas linking cheetah populations between Botswana and Namibia (Winterbach et al., 2015). The study area focussed on the commercial farming block made up of more than 200 cattle or game farms. The habitat is primarily made up of low tree and shrub savannah and receives low annual rainfall (400 mm/year). Most water in the area is artificially pumped via boreholes or found in natural pans during the rainy season with no permanent surface water (Cole and Brown, 1976). For further description see Kent (2011) and Houser et al. (2009b).

### Animal collaring

Approval for the research study was provided by the Botswana Ministry of Environment, Wildlife and Tourism and Department of Wildlife and National Parks on permit number EWT8/36/4. The work was approved by the RVC Ethics and Welfare Committee (LOCATE 2013-1233).

Cheetahs in the Ghanzi farmlands have been monitored by researchers for several years through the use of camera traps at identified marking trees. This long-term monitoring has allowed researchers to determine whether cheetahs observed at these locations are resident or non-resident individuals. Seven cheetahs were fitted with high-time resolution GPS collars by a veterinary specialist in August 2014 with a drop off date for February 2015. Data were collected for an average of 128 days per individual (range 77-189 days). Two cheetahs were single males, three were from a coalition of four and two were individuals from coalitions of two. From the seven collared cheetahs only four were used for analysis; one single male (M3), two from separate coalitions of two (M1 and M4) and one from the coalition of four (M2). As the three collared males from the coalition of four stayed within 100 m of each other for 80% of the GPS fixes (Hubel et al., 2016a) these were considered non-independent; one collar from a single male failed to collect data after two weeks.

### Collar configuration

Royal Veterinary College (RVC) high-time resolution GPS collars were designed to collect data to study movement patterns and hunting behaviour. The collars contain a GPS receiver which provides highly accurate positional data. This enables the cheetahs' movements and activity to be tracked over time and space in great detail. In order to manage power consumption effectively, the collars were set to switch dynamically between three different operating states depending on the animal's activity level (Hubel et al., 2016b; Wilson et al., 2013). Activity levels were based on accelerometer measurements. At low activity, when the animal was deemed to be resting, hourly fixes were recorded; with increased activity, the sample rate increased to 5 min fixes. When the animal was judged to be running the GPS rate increased to 5 Hz. The data were retrieved by downloading via external download boxes placed at marking trees or directly from the collar when retrieved following pre-timed automatic drop-off (Sirtrack Ltd., Havelock North, New Zealand). In this study, the main objective was to study movement patterns utilising GPS points recorded by the collars, but not all of the high-sample rate of points was required. As such, the number of points used were adjusted depending on the particular analysis as described below.

### Home range analysis

Home range analysis using minimum convex polygons (MCP) (Jenrich and Turner, 1969) and fixed kernel density estimates (KDE) were calculated in R (R Core Team, 2015) using the *adehabitat* package (Calenge, 2015). Calculation of MCPs at 50% and 95% used all GPS data points collected per individual. For KDE of home range at 50% and 95%, we used just a single daily GPS fix chosen closest to 12:00AM to meet the independence of data assumption. Smoothing parameters for KDE analysis were first tested using least-squares cross validation (LSCV) which gives the least biased estimates (Seaman and Powell, 1996), but as they did not converge in all cases, we adjusted our analysis to using the reference bandwidth parameter (href). However, as the href can often oversmooth data, we chose to use 80% of the href value as a better estimate of smoothing (Kie et al., 2010). GPS locations were incorporated into ArcMap 10.3.1 (ESRI, Redlands, CA, USA) to generate home range maps.

### Calculation of visitation and duration rates

The R package T-LoCoH (Lyons et al., 2013) was used to calculate two dependent variables: visitation rate (number of separate visits to a hull) and duration of visits (mean number of points per hull) using GPS locations recorded by each collar. All GPS points were used in each data set to check for outliers and adjusted to local time. GPS points were then thinned to one per hour to maintain consistency of points across each individual. T-LoCoH creates a minimum convex hull around each data point combining both temporal and spatial distances between two points. A time-scaled dependent (TSD) variable *s* is determined based on balancing both time and space. Low values of *s* indicate time having little importance. To determine *s* we plotted different values of *s* over a range of different time values (range 12-96 h). *s* values were chosen at 24 h periods as time and space were mostly equalised in all cases. Time-based local convex hulls can be created using the *k* method (set number of nearest neighbour points), *r* method (nearest neighbours in a radius *r*) or *a* method (both time and distance are incorporated). We chose the *a* method as this can take into account areas where points may be thin and scattered, for example when an individual may enter areas not commonly used (Lyons et al., 2013). Creation of local convex hulls was completed by following methods described in Lyons et al. (2013) and the associated Tutorial and Users Guide. Although collars were set to record at regular time intervals, there were irregularities in satellite acquisitions between collars, as well as length of collaring periods, which led to differences in the number of fixes per individual. Therefore we generated *a* values separately for each cheetah. Local convex hulls were sorted by time-use metrics using an inter-visit gap (IVG) period of 12 h and this was used to create independent visitation and duration rates. Time-based local convex hulls can also be used to generate home ranges. Taking into account the temporal nature of location data can be argued to be a more suitable method for correlated data points (Fieburg and Börger, 2012; Lyons et al., 2013), we also generated home range estimates for comparison with traditional estimates using MCP and KDE methods as described above.

### Incorporation of landscape features

**Fig. 3.**
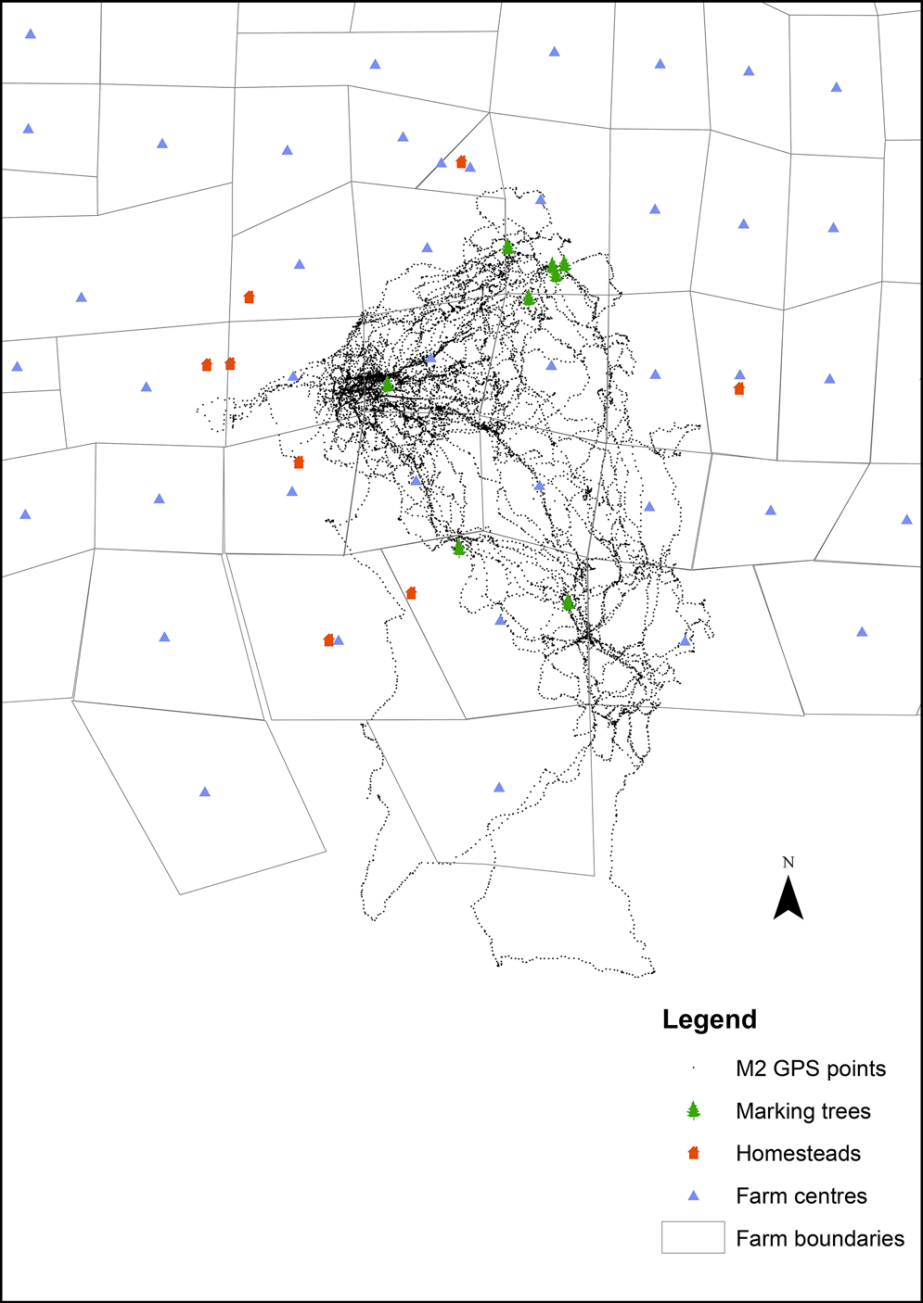
**GPS locations of one collared male cheetah (M2) *Acinonyx jubatus*, scent-marking trees, homesteads and the centre point of farms, in a farming area of western Botswana.**

Shape files that recorded previously identified marking trees, homesteads, centre points of farms and farm type were incorporated into ArcMap (Fig. 3) as spatial variables of interest. Other potential influential features such as bore holes and roads were not available for the majority of farms used and were not included in this study. We calculated the distance of each GPS point to the nearest of each of the variables. As we did not know all possible marking trees or homesteads in the study area, we restricted the data set to a maximum range of 6 km for each for these variables. This distance represents an average daily travel distance for males in this area (Houser et al., 2009a), which allowed us to look at the effect of avoidance or selection when cheetahs were within an average daily distance from each feature. Mixed farms (cattle and game) represented only a small fraction of the farms in the area and were removed from our analysis to increase power and simplify the analysis.

### Data analysis

As GPS data generates multiple response outputs per individual, it is necessary to account for correlation in data points per individual. Mixed-effect models using subject as a random factor can account for correlated data points, however it is not recommended for random effects in which the number of levels is less than five (Bolker et al., 2009). Therefore, we chose to use a simpler two-stage approach that is effective in modelling correlated data and applicable to our large datasets (Fieberg et al., 2010; Gelman, 2005; Murtaugh, 2007). In R we used the package *lme4* (Bates et al., 2015) to perform linear (LM) and generalised linear (GLM) regression models to analyse the relationship of duration and visitation rate to a set of three distance-related predictor variables (distance to marking tree, distance to homestead, distance to centre of farm) and also farm type. As we had subset our data to a maximum of 6 km distances (for the distance variables), this effectively removed some data points in the other two predictors. As we did not wish to lose valuable information per variable, we chose to run our models separately for each fixed factor. However, we also ran a global model incorporating all the distance-related variables to determine whether we still obtained similar results despite the lower sample size. For stage 1, each of the four cheetahs were modelled separately for each of the fixed factors. Each model was checked for normality and deviances from homoscedasticity were checked by visual inspection of residual plots. The response variable duration was log transformed where necessary. As visitation is a count variable we used the Poisson distribution, with a log-link function. Overdispersion was checked using the *AER* package (Kleiber and Zeileis, 2008) and in these cases we corrected the standard errors using a quasi-GLM model. We created vectors of model coefficients and a block variance-covariance matrix for each of the two predictors. In stage 2 we used the package *metafor* (Viechtbauer, 2010) to conduct a multilevel meta-analysis using the model coefficients (intercept and slope) against each of the response variables. A random effect of subject of both intercept and slope was also added to the model. Coefficients were allowed to be heterogeneous and correlated using an unstructured variance-covariance matrix. The Q statistic was used to test whether effect sizes between subjects are homogenous using the chi-square distribution. A τ^2^ statistic is produced accounting for the amount of variability among effect sizes not accounted for by the predictor variable. τ^2^ values equal to zero in mixed-effect models imply that heterogeneity of true effect sizes are due to the predictor in the model (Viechtbauer and Cheung, 2010). Models were checked by plotting standardised residuals and checking for influential outliers using the method described by Habeck and Schultz (2015), whereby values whose standardised residuals were greater than three and hat values greater than two times the mean hat value were considered as potential outliers. Significance was set at *P*<0.05 and means are reported with standard errors unless specified elsewhere. In log transformed models and models using the Poisson distribution, coefficients and 95% confidence intervals were back-transformed, and these results are used in all graphs and tables presented.
